# Mr. Armstrong, on Inoculation

**Published:** 1806-03

**Authors:** S. L. Armstrong

**Affiliations:** St. Cross, near Winchester


### To THOMAS PAYTHERUS, Esq.

THE following Cases, on the subject of Inoculation, appearing to me rather singular, I have taken the liberty of laying them before you, for the following reason, that tho' they may contain nothing new to the medical world, yet, nevertheless, they offered to my mind a most satisfactory proof of the efficacy of the vaccine mode of inoculating.

### *The Case of Lydia Carter, now Barnet.*

About nine years ago, Lydia Carter lived in the service of George Ricketts, Esq. of Candover, in this county. Having never had the small-pox, she was inoculated with vaccine matter, and had the disorder in the usually mild way. About two years subsequent, she married, and came to settle in the village where I live, her husband being a miller's labourer. At this period her brother, James Carter, lived in the adjacent village of Twyford, having a wife and five children. A benevolent lady of that neighbourhood had, about two years before, recommended him to have himself, wife, and five children, inoculated with the vaccine matter. He unfortunately having no care for himself, rejected it, but permitted his wife and children to be inoculated after the vaccine method. They had the disease in its usually mild form, and the father experienced no inconveniency. In two years subsequent to this, he himself sickened in the natural small-pox, and had it in a most violent degree. At this period Lydia Barnet went down to Twyford, and attended her brother night and day for a week, and was exposed to every possible degree of infection. A medical gentleman of the first abilities was called in; not having himself inoculated the wife and children of this family, but only understanding that they had been inoculated, he could not answer for it that they had had the disease; he therefore proceeded to inoculate them with the natural matter. As for Lydia Barnet, as she had been a vaccine patient of his own when at Mr. Ricketts's, he refused to inoculate her; she was, however perfectly secure, and the wife and children of James Carter were equally so, as the inoculation of them from the natural matter had no effect whatever. After the lapse of two years more, and four from the inoculation of Lydia Barnet with the vaccine matter, the natural small-pox broke out at the mill where her husband's daily employment required his presence. There were five people of different ages down at the same time. Upon this, Lydia Barnet, still apprehensive she might be infected, submitted to be inoculated with natural matter; and the person who inoculated her (being no advocate for the vaccine mode) boasted with confidence that he would communicate the disease. In this, however, he was completely foiled, and was by this experiment convinced of the efficacy of a system, which, previous to this, he had affected to despise. Lydia Barnet is still my neighbour, and adds her testimony to the above case.

*Case of Mrs. Clewer and her Son.*

John, son of Mrs. Clewer, a neighbour of mine, a boy at this time of twelve years of age, was inoculated with his mother about eleven years ago, from matter taken from the small-pox in the natural way. The child being then at the breast, was inoculated three days before the mother. The mother had the disease in a favorable way; the child did not appear to sicken, but his arm rose very kindly, and every thing appeared to be well, and no doubt was entertained by the parents at least, but that they were both secure. Last summer two years, Sarah, a second child of Mrs. Clewer, was inoculated with the small-pox; she was then seven years of age; she had the disease very favourably, but what is remarkable, her brother, whom his mother had suckled under inoculation, and who had been inoculated himself along with her, took the infection from his sister in the natural way, and had the disease so bad that he was covered from head to foot. The parties are all now living in St. Cross.

1st. Quere, Had a case similar to this happened under the vaccine inoculation, would it not have injured the practice in a great degree in the vicinity where it happened?

2d Quere, The first disease in the child's arm being evidently a spurious one, is it not probable, that where infection fection ever follows the vaccine inoculation, it must be owing to an *apparent* but not real communication of the disease in the first instance?

Deduction from both cases, considered in conjunction. It appears that the true vaccination is proof against every infection of the small-pox, whether natural or attempted to be communicated, and that it is not more liable to fail than the small-pox by inoculation either is or ever has been.

*Case of William Cole and Sarah Fry.*

Sarah Fry, born in the year 1739, lived as house-keeper with William Cole, aged then eighty-two, in this village. She was seized with the natural small-pox in the year 1803. On the discovery of this, medical assistance being called in, the gentleman that attended hesitated to vaccinate the old man, William Cole, fearing lest he might have taken the infection previously in the natural way; but nevertheless, in compliance with the old man's wishes, Sarah Fry was removed, and he was inoculated with vaccine matter; his arm rose well, and he is now a living witness of the utility of vaccination even at the latest period of life, being at this time 86 years of age. Sarah Fry is also recovered, and is still living, aged 67. Should the above cases be of any service I shall be extremely happy. They are written, as you may perceive, currente calamo, for truth needs no ornament.

*St. Cross, near Winchester,* I am, &c.

*January* 15, 1806. S. L. ARMSTRONG.

